# Aberrant Functional Connectivity and Brain Network Organization in High-Schizotypy Individuals: An Electroencephalography Study

**DOI:** 10.1093/schbul/sbaf004

**Published:** 2025-02-04

**Authors:** Jelena Trajkovic, Giulia Ricci, Gabriele Pirazzini, Luca Tarasi, Francesco Di Gregorio, Elisa Magosso, Mauro Ursino, Vincenzo Romei

**Affiliations:** Centro studi e ricerche in Neuroscienze Cognitive, Dipartimento di Psicologia, Alma Mater Studiorum – Università di Bologna, Campus di Cesena, Cesena 47521, Italy; Department of Cognitive Neuroscience, Faculty of Psychology and Neuroscience, Maastricht University, Maastricht 6229 ER, The Netherlands; Department of Electrical, Electronic, and Information Engineering “Guglielmo Marconi,” Alma Mater Studiorum – Università di Bologna, Campus di Cesena, Cesena 47521, Italy; Department of Sleep and Dreams, Netherlands Institute for Neuroscience, Institute of the Royal Netherlands Academy of Arts and Sciences, Amsterdam 1105 BA, The Netherlands; Department of Electrical, Electronic, and Information Engineering “Guglielmo Marconi,” Alma Mater Studiorum – Università di Bologna, Campus di Cesena, Cesena 47521, Italy; Centro studi e ricerche in Neuroscienze Cognitive, Dipartimento di Psicologia, Alma Mater Studiorum – Università di Bologna, Campus di Cesena, Cesena 47521, Italy; Centro studi e ricerche in Neuroscienze Cognitive, Dipartimento di Psicologia, Alma Mater Studiorum – Università di Bologna, Campus di Cesena, Cesena 47521, Italy; Department of Electrical, Electronic, and Information Engineering “Guglielmo Marconi,” Alma Mater Studiorum – Università di Bologna, Campus di Cesena, Cesena 47521, Italy; Department of Electrical, Electronic, and Information Engineering “Guglielmo Marconi,” Alma Mater Studiorum – Università di Bologna, Campus di Cesena, Cesena 47521, Italy; Centro studi e ricerche in Neuroscienze Cognitive, Dipartimento di Psicologia, Alma Mater Studiorum – Università di Bologna, Campus di Cesena, Cesena 47521, Italy; Facultad de Lenguas y Educación, Universidad Antonio de Nebrija, Madrid 28015, Spain

**Keywords:** schizotypy, Granger causality, graph theory, electroencephalography, resting-state networks, brain rhythms

## Abstract

**Background and Hypothesis:**

Oscillatory synchrony plays a crucial role in establishing functional connectivity across distinct brain regions. Within the realm of schizophrenia, suggested to be a neuropsychiatric disconnection syndrome, discernible aberrations arise in the organization of brain networks. We aim to investigate whether the resting-state functional network is already altered in healthy individuals with high schizotypy traits, highlighting the pivotal influence of brain rhythms in driving brain network alterations.

**Study Design:**

Two-minute resting-state electroencephalography recordings were conducted on healthy participants with low and high schizotypy scores. Subsequently, spectral Granger causality was used to compute functional connectivity in *theta*, *alpha*, *beta*, and *gamma* frequency bands, and graph theory metrics were employed to assess global and local brain network features.

**Study Results:**

Results highlighted that high-schizotypy individuals exhibit a lower local efficiency in *theta* and *alpha* frequencies and a decreased global efficiency across *theta*, *alpha*, and *beta* frequencies. Moreover, high schizotypy is characterized by a lower nodes’ centrality and a frequency-specific decrease of functional connectivity, with a reduced top-down connectivity mostly in slower frequencies and a diminished bottom-up connectivity in faster rhythms.

**Conclusions:**

These results show that healthy individuals with a higher risk of developing psychosis exhibit a less efficient functional brain organization, coupled with a systematic decrease in functional connectivity impacting both bottom-up and top-down processing. These frequency-specific network alterations provide robust support for the dimensional model of schizophrenia, highlighting distinctive neurophysiological signatures in high-schizotypy individuals.

## Introduction

Schizophrenia represents one of the most debilitating and complex neuropsychiatric disorders. Along with heterogeneous positive and negative clinical symptoms, it also causes impairments in various cognitive domains, such as perception, attention, and executive functions.^1,2^ It is often understood as a disconnection syndrome, physiologically expressed by abnormal functional connectivity between brain regions, and resulting in a disruption of information integration.^3^ Substantial evidence supports the disconnection hypothesis, emphasizing that schizophrenia is characterized not merely by focal changes, but rather by widespread and pattern-like disturbances across various brain areas.^4–6^ Since it was first postulated, more than 20 years ago, and thanks to the methodological and computational advances, this hypothesis has gained significant support.^7^

Exploring alterations in functional connectivity can significantly enhance our understanding of neural mechanisms underlying schizophrenia, offering potential neurophysiological markers for this condition.^8^ Graph theory provides a powerful framework for conceptualizing the complex system of the brain as a network of interconnected nodes, revealing differences in the schizophrenic network architecture through global and local graph-related metrics.^9^ Global metrics define brain network organization, delineating a tendency toward information segregation—identifying densely interconnected clusters of brain regions—and information integration—measuring the efficiency of information transmission. In healthy individuals, maintaining the balance between segregation and integration is crucial for adapting to a dynamic environment.^10^ However, in mental illnesses like schizophrenia, an imbalance in these dynamics may be present. Structural connectivity studies on schizophrenia reveals less efficient organization, highlighted by lower interconnection of neighboring nodes^11^ and longer paths for information transmission in frontal and temporal regions.^11^ Moreover, compromised functional connectivity has been identified across various brain systems, including auditory, default mode, self-referential, and somatosensory networks,^12^ as revealed through magnetic resonance imaging (MRI), magnetoencephalography (MEG), and electroencephalography (EEG) studies.^13–15^

Importantly, the clinical and potentially physiological traits of schizophrenia are evident not only in diagnosed individuals but also in subclinical populations, their relatives, and during prodromal phases, although less severe. There is a growing consensus that psychosis exists on a continuum ranging from subclinical experiences in the general population, defined as schizotypy, to clinical symptoms of schizophrenia.^16–18^ Schizotypy can be defined as a personality organization reflecting underlying vulnerability associated with an elevated risk of developing psychosis.^19^ A comprehensive understanding of shared physiological mechanisms across schizotypy dimensions can support the development of early detection strategies and preventive interventions for high-risk populations.^17^

The present study seeks to deepen our understanding of the spontaneous neural mechanisms underlying individuals with high schizotypy by exploring the functional network features across various oscillatory frequencies using a data-driven approach. Resting-state EEG was analyzed to capture intrinsic connectivity and explore the role of neural synchronization in brain functioning. To assess connectivity patterns between brain regions, we used spectral Granger causality (GC), selected for its ability to effectively infer directional and frequency-dependent functional connectivity, providing a detailed representation of brain network organization.

We investigated different topological features of brain networks, including global and local network characteristics. Fully supported by previous coherent and robust research in schizophrenia patients,^20–31^ here we wanted to add a fundamental layer of analysis, by showing similar patterns in a nonclinical population with high schizotypy traits. This is supported by findings of altered functional brain connectivity in antipsychotic-naive first-episode psychosis^32^ and individuals with psychotic-like experiences,^33^ but also schizotypy.^34,35^ Indeed, schizotypy scores have been found to predict lower functional connectivity across different brain networks,^36–38^ giving rise to the hypothesis that the development of schizotypy may be related to changed brain network connectivity.^39^ Therefore, here we expect a reduction in widespread functional brain connectivity in individuals with high levels of schizotypy compared to individuals with low levels of schizotypy. Based on our biobehavioral model of schizophrenia spectrum^40^, and frequency specificity of top-down and bottom-up signals, we expect fronto-posterior (top-down) connectivity across lower frequencies and posterior-frontal (bottom-up) connectivity across faster frequencies to be altered in schizotypy.

## Methods

### Participants

One hundred nine healthy participants took part in the study (67 female, mean age = 23.26 years, SD = 3.41, see also Table 1), which was conducted in accordance with the Declaration of Helsinki and approved by the Bioethics Committee of the University of Bologna. Each participant signed a written informed consent before taking part in the study, and all data were analyzed and reported anonymously. Participants were selected on a sample of 750 students from the University of Bologna based on the presence of schizotypal traits, estimated via Schizotypal Personality Questionnaire (SPQ). SPQ was developed to assess schizotypal personality traits or proneness to psychosis in the general population, where scores in the top 10% of SPQ scores fulfilled a clinical diagnosis of schizotypal personality disorder (SPD). Thus, the SPQ may be useful in screening for SPD in the general population and also in researching the correlates of individual schizotypal traits. The 3-factor model measured by the SPQ, consisting of cognitive-perceptual, interpersonal, and disorganized factors, was shown to fit the data in patients with schizophrenia, as well as in the SPD and nonclinical population,^41^ giving support to the hypothesis that the SPQ reflects the genetic vulnerability to schizophrenia.^42^ This supports the dimensional model of schizotypy, ranging from the general population to SPD and patients with schizophrenia. Therefore, SPQ represents a gold standard in schizophrenia/schizotypy research, with schizotypy traits both in the general population and schizophrenia patients negatively correlating with brain connectivity metrics.^36–38^ Subsequently, 2 age-matched (*M*_high_ = 23.78, *M*_low_ = 22.75, *t*(101) = 1.548, *P* = .124) and gender-matched (χ^2^ = 0.506, *P* = .477) groups were created: a Low Schizotypal Group (LSG, Nl*N*_*l*_ = 54) with scores below the 20th percentile (*M* = 7.83, SD = 3.09) and a High Schizotypal Group (HSG, *N*_*h*_ Nh= 55) with scores above the 80th percentile (*M* = 41.40, SD = 6.44) who agreed to take part in the present study (for more details about the subject- and group-level variability, please see Supplementary Figure 1).

**Table 1.** Descriptive Statistics

	Age		SPQ		Gender	
	Low	High	Low	High	Low	High
Valid	54	55	54	55	54	55
Mean	23.784	22.785	7.833	41.400	64% (F)	58% (F)
SD	3.743	3.002	3.094	6.448		

Abbreviations: F, female; SPQ, Schizotypal Personality Questionnaire.

### EEG Acquisition and Preprocessing

Participants were comfortably seated in a room with dimmed lighting conditions. During the experiment, their EEG activity was recorded while they kept their eyes closed for a duration of 2 minutes, a duration proven to be long enough to obtain robust estimates of intrinsic brain activity.^43–48^ A 64-electrode cap was positioned following the international 10-10 system. EEG signals were recorded at a rate of 1000 Hz and referenced to the right mastoid, while the impedance of all electrodes was maintained below 10 kΩ. Post-recording, we conducted an offline EEG data processing using custom MATLAB scripts (version R2021b) in conjunction with the EEGLAB toolbox.^49^ First, we down-sampled the data to 500 Hz and applied a notch filter (50 Hz) and a bandpass filter (0.5-60 Hz). Subsequently, bad channels were identified by computing the correlation coefficient between each electrode and the others^50^ and then interpolated with the neighboring channels. On average, each participant exhibited 0.5 (SD: 1.1) bad channels. Following bad channel correction, we re-referenced the EEG recordings to the mean of all electrodes. This method is widely accepted in EEG source localization for its ability to standardize signals and minimize model errors in the forward model, resulting in more accurate and reliable inverse source estimations.^51,52^ Lastly, Independent Component Analysis (ICA) was employed for the removal of EEG artifacts. Overall, 9.3 ± 3.6 independent components were removed for each participant.

### Cortical Sources Reconstruction and Regions of Interest Definition

Cortical source activity was reconstructed starting from the preprocessed EEG signals. The Brainstorm Matlab toolbox^53^ was employed to compute intracortical current densities. A template 3-layer head model comprising the scalp, the outer skull surface, and the inner skull surface (ICBM152 MNI template) was used to address the forward problem. This model encompassed a discretized cortical source space featuring 15 002 vertices. Solving the forward problem involved the Boundary Element Method within the OpenMEEG software.^54^ For estimating cortical sources, we adopted the standardized Low-Resolution Electromagnetic Tomography (sLORETA) algorithm, a linear inverse solution method for 3D EEG distributed source modeling.^55^ The algorithm computes a weighted minimum norm solution, where localization inference relies on standardized current density estimates. The resulting solution is instantaneous, distributed, discrete, and linear and is presented with zero dipole localization error under ideal (noise-free) conditions. We considered constrained dipole orientations, disposed perpendicularly to the cortical surface. For each participant, we extracted the resting-state time series of standardized current densities of all 15 002 cortical vertices. Subsequently, the cortical vertices were grouped based on the Desikan-Killiany atlas^56^ available in Brainstorm. This atlas defines 68 regions of interest (ROIs), as described in Table 2. Within each ROI, the activities of vertices were averaged at each time point, thereby yielding a singular time series representative of the cortical ROI’s activity.

**Table 2.** Brain region categories based on the Desikan-Killiany atlas and their labels

ROI	Label	Lobe	ROI	Label	Lobe
Banks of superior temporal sulcus	BK	Temporal	Parahippocampal	PH	Temporal
Caudal anterior cingulate	cAC	Frontal	Pars opercularis	pOP	Frontal
Caudal middle frontal	cMF	Frontal	Pars orbitalis	pOR	Frontal
Cuneus	CU	Occipital	Pars triangularis	pTR	Frontal
Entorhinal	EN	Temporal	Pericalcarine	PCL	Occipital
Frontal pole	FP	Frontal	Postcentral	POC	Parietal
Fusiform	FU	Temporal	Posterior cingulate	PCG	Parietal
Inferior parietal	IP	Parietal	Precentral	PRC	Frontal
Inferior temporal	IT	Temporal	Precuneus	PCU	Parietal
Insula	IN	Parietal	Rostral anterior cingulate	rAC	Frontal
Isthmus cingulate	IST	Parietal	Rostral middle frontal	rMF	Frontal
Lateral occipital	LO	Occipital	Superior frontal	SF	Frontal
Lateral orbitofrontal	lOF	Frontal	Superior parietal	SP	Parietal
Lingual	LG	Occipital	Superior temporal	ST	Temporal
Medial orbitofrontal	mOF	Frontal	Supramarginal	SMG	Parietal
Middle temporal	MT	Temporal	Temporal pole	TP	Temporal
Paracentral	PAC	Frontal	Transverse temporal	TT	Temporal

Abbreviation: ROI, regions of interest.

### Functional Connectivity Through Frequency-Domain GC

To investigate the potential brain networks alterations underlying high and low schizotypy traits, we computed directed functional connectivity within the *theta* (4-8 Hz), *alpha* (8-12 Hz), *beta* (14-30 Hz), and *gamma* (30-40 Hz) frequency bands among the 68 reconstructed cortical ROIs. The frequency-domain GC estimator was employed for the computation of functional connectivity, which yields weighted and directional metrics of the causal interactions between ROIs.

In general, the GC estimator is based on an autoregressive (AR) modeling framework. Given 2 time series *x*_*k*,*i*_[*n*] and *x*_*k*,*j*_[*n*] (where n*n* denotes discrete time) representing the activity at 2 distinct brain regions (ROI_*i*_ and ROI_*j*_) for participant k*k*, the GC estimator quantifies the causal interaction from ROIiROI_*i*_ to ROIjROI_*j*_. This estimate arises from the enhancement in predictingxk,j[n] *x*_*k*,*j*_[*n*] using a bivariate AR model (incorporating past values of both *x*_*k*,*j*_ and *x*_*k*,*i*_) versus a univariate AR model (relying solely on past values of *x*_*k*,*j*_) at a certain model order *p*. According to the Geweke’s approach (1982, 1984)^57^, the power spectrum of *x*_*k*,*j*_[*n*] can be partitioned into “intrinsic” and “causal” components, with the latter predicted by *x*_*k*,*i*_[*n*].^58^ At each frequency *f*, the spectral GC from ROIiROI_*i*_ to ROIjROI_*j*_ is defined as the logarithm of the ratio between the total power spectrum of *x*_*k*,*j*_[*n*] and the difference between the total power spectrum and the “causal” power predicted by *x*_*k*,*i*_[*n*]. Consequently, at a specific frequency *f*, the estimated value *GC*_*i→j*_(*f*) increases (>0) as the causal power increases.

For each participant *k*, the frequency-domain GC was computed for all ROI pairs and in both directions. Moreover, the order *p* of the AR models was set to 30, based on a previous analysis,^59–61^ which indicated that GC values remain relatively stable for *p* ≥30.

The frequency-domain GC computation provided a 68 × 68 connectivity matrix (with all auto-loops equal to zero) for each frequency sample (*n* sample = 2501, frequency resolution = 0.1 Hz). Then, the spectral connectivity matrices were averaged within the *theta*, *alpha*, *beta*, and *gamma* bands, obtaining a 68 × 68 connectivity matrix for each frequency band and participant.

Subsequently, we derived sparse connectivity matrices via statistical comparison between HSG and LSG, in order to reduce the noise, a procedure commonly used to remove noise and spurious connections.^62,63^ For this step, for each band, a 2-tailed nonparametric permutation *t*-test (5000 permutations) was employed, and significant connections were defined as having an uncorrected *P*-value lower than .05. Consequently, among the possible 68 × 68 connections, solely the significant connections between HSG and LSG were retained for each frequency band and participant, while all nonsignificant connections (uncorrected *P*-value higher than .05) were set to zero. It should be noted that the results presented in the figures were obtained from these sparse matrices. Moreover, we have replicated the analyses performed on the sparse matrices on the complete (non-threshold) connectivity matrices, as presented in Supplementary Materials (Supplementary Figures S2i–iii).

### Graph Theory Indices

The interconnections among different cortical ROIs can be depicted as a weighted graph.^64^ In this graphical representation, the strength of connectivity between any 2 ROIs is denoted by the edge’s weight, while the ROIs are the graph’s nodes. This approach has the advantage of examining brain connectivity circuits as if they were networks, thereby enabling the use of graph theory measures.^65,66^

In this study, we aimed at investigating the brain networks in LSG and HSG using metrics from graph theory and network analysis. Our analysis firstly focused on 2 fundamental aspects of global brain network organization: integration and segregation tendency. Then, we extracted local measures of graph theory in order to identify the cortical ROIs which exhibit the main differences in terms of information outflow and inflow, and their resulting network. Finally, we pooled together the networks resulting from the previous analysis and defined a fronto-posterior and a posterior-frontal connectivity index to quantify the different top-down and bottom-up brain mechanisms in HSG and LSG.

### Global Network Indices

Within the domain of brain network analysis, the evaluation of global parameters holds pivotal significance, offering profound insights into the network functionality. Indeed, these parameters provide a comprehensive understanding of the brain network’s ability to balance efficient long-range communication and specialized local processing. The integration tendency refers to the network’s efficiency in transferring information across long distances. Conversely, segregation tendencies evaluate the presence of densely interconnected clusters or communities embedded within the network’s architecture. In sum, in graph theory, “efficient organization” is used to describe a network that enables a rapid integration of information from local, specialized brain areas (ie, high local efficiency [*LE*]) even when they are distant (ie, higher global efficiency [*GE*]). These aspects are crucial in identifying the alterations characterizing schizotypy.

In this study, we employed *GE* to assess the brain network’s integration tendency. This metric evaluates how effectively information is exchanged across all nodes, reflecting the network’s ability to integrate information over long distances. In a weighted network, such as in our case, edges with higher weights correspond to shorter distances, meaning the shortest path between 2 nodes is determined by the path that maximizes the sum of the weights (minimizing distance). Additionally, in directed graphs, the shortest path may vary based on the direction of connections. Mathematically, *GE* is the average of the inverse of the weighted shortest path lengths between all node pairs. Shorter paths, which indicate stronger connectivity, result in higher efficiency. Therefore, a higher *GE* signifies more effective communication across distant regions of the network, highlighting how well-connected pathways facilitate information flow.

Conversely, *LE* serves as a metric to assess the degree of brain network segregation. *LE* quantifies a network’s fault tolerance by evaluating whether communication between neighboring nodes remains efficient when one node is removed. A higher *LE* indicates greater local robustness, reflecting the ability of specific regions to maintain communication despite disruptions. Mathematically, *LE* is the average of the *GE* of subgraphs formed by each node’s neighbors. For each node, a subgraph is constructed by removing that node and calculating how efficiently its neighbors communicate with one another. This average provides a measure of how well local regions sustain efficient communication, even in the absence of individual nodes.^67^

For the computation of these metrics, we relied upon the Brain Connectivity toolbox in Matlab,^67^ which proved instrumental in extracting global brain network features. Starting from the sparse 68 × 68 matrices, we computed *LE* and *GE* indices for each subject and frequency band. Subsequently, we employed linear mixed models (LMMs), considering subjects as a random factor, to analyze the effects of group (LSG and HSG), frequency band (*theta*, *alpha*, *beta*, and *gamma*), and their interaction in predicting *LE* and *GE*. Next, we conducted planned comparisons using 2-tailed Student’s *t*-tests and corrected for multiple comparisons employing Bonferroni method.

### Local Network Indices

Local brain network indices such as centrality are pivotal tools for assessing the influence of specific cortical regions within the complex neural architecture. The assessment of nodes’ centrality shows how certain nodes serve as keystone elements in shaping brain functioning. Two key local centrality indices in directed networks are Outdegree centrality and Indegree centrality. These 2 indices assess how cortical regions within the brain network, respectively, propagate outward information and receive inflow information. In the following, the symbol *A* denotes a generic adjacency matrix containing all edge weights. Specifically, the matrix element *A*_*ij*_ represents the weight of the directed edge connecting node *i* to node *j*.

Outdegree centrality quantifies the influence of each node on all the other nodes of the network and is defined as the summation of edge weights originating from a given node. Mathematically, Outdegree is expressed as:


$$\begin{array}{l} Outdegree_{i}=\underset{j}{\sum }\;A_{i,j} \end{array}$$

Indegree centrality, on the other hand, quantifies the extent to which the node is the target of the information flow from other nodes in the network and is defined as the summation of edge weights entering a particular node. Mathematically, Indegree is expressed as:


$$\begin{array}{l} Indegree_{i}=\underset{j}{\sum }\;A_{j,i} \end{array}$$

These local network metrics were calculated using the function offered by Matlab’s libraries found within the “Graph and Network Algorithms” category (Matlab R2021a). Due to their direct reliance on the strength of incoming and outgoing connections, Indegree and Outdegree offer immediate insights into nodes that are most actively engaged in the transmission (Outdegree) and reception (Indegree) of information.

For each participant, we started our analysis with the sparse 68 × 68 matrix. Subsequently, we calculated both Outdegree and Indegree centrality indices for each frequency band and for the 68 cortical ROIs and identified the ROIs that significantly differed between HSG and LSG. The significance of a ROI was determined by applying the 2-tailed nonparametric permutation *t*-test (5000 permutations) and considering Bonferroni-corrected *P*-values lower than .05. Then, once the significant cortical ROIs for each centrality metric and frequency band were identified, we visually represented the differences between the LSG and HSG, by plotting the brain network differences. We first displayed the differences among connections originating from nodes with significant Outdegrees (Figure 2), and then the differences in connections entering nodes with significant Indegrees (Figure 3). This approach allowed us to highlight variations between the 2 groups in terms of connectivity patterns.

**Figure 1.**
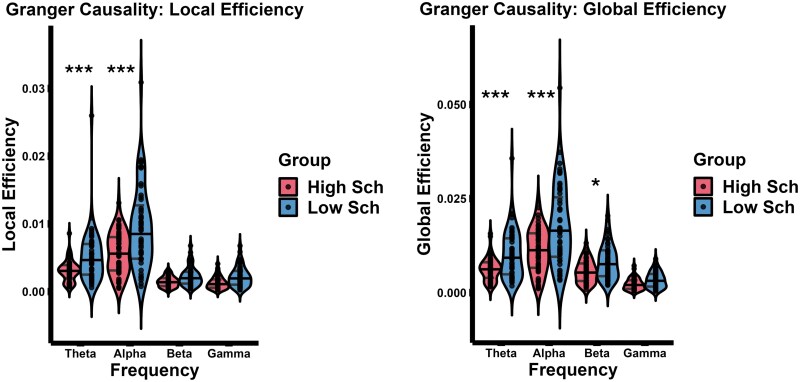
Global Network topology indices. Left panel shows the local efficiency (mean ± SEM) for Low-Schizotypy Group and High-Schizotypy Group and for *theta*, *alpha*, *beta*, and *gamma* frequency bands. Right panel shows the global efficiency (mean ± SEM) for Low-Schizotypy Group and High-Schizotypy Group and for *theta*, *alpha*, *beta*, and *gamma* frequency bands.

**Figure 2.**
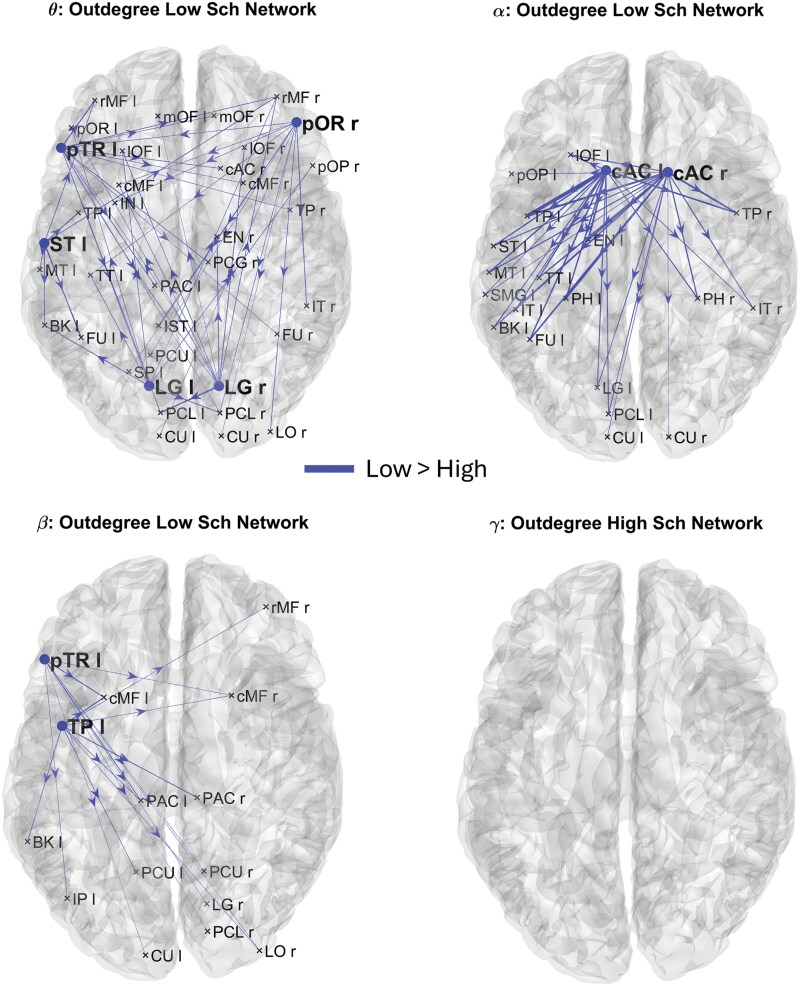
Brain networks resulting from connectivity differences exiting from the cortical ROIs which exhibited significant Outdegree differences between groups in *theta* (A), *alpha* (B), *beta* (C), and *gamma* (D) frequency bands. The lines and nodes denote connections and Outdegree higher in the Low Schizotypy compared to the High Schizotypy. There are no lines and nodes denoting connections and Outdegree higher in the High Schizotypy compared to the Low Schizotypy. The plotted connections run from the cortical ROIs with significant Outdegree (marked with a dot) toward generic input ROIs (marked with a cross). The thickness of each link varies according to the value of the connection difference.

**Figure 3.**
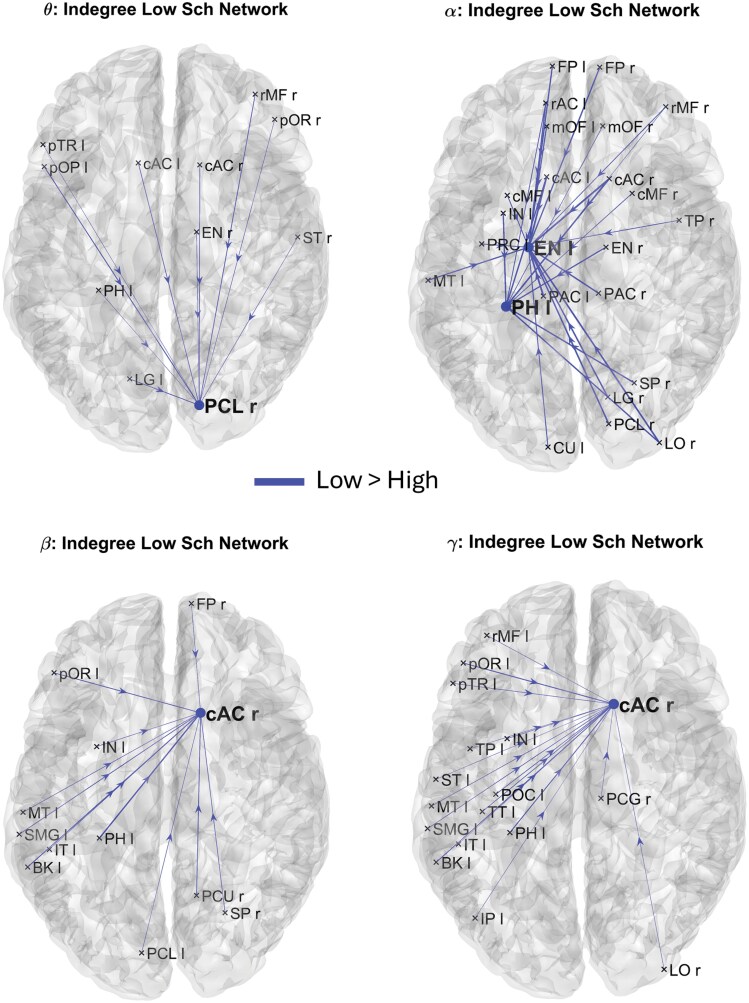
Brain network resulting from connectivity differences entering to the cortical ROIs which exhibited significant Indegree differences between groups in *theta* (A), *alpha* (B), *beta* (C), and *gamma* (D) frequency bands. The lines and nodes denote connections and Indegree higher in the Low Schizotypy compared to the High Schizotypy. There are no lines and nodes denoting connections and Indegree higher in the High Schizotypy compared to the Low Schizotypy. The plotted connections run from a generic output ROI (marked with a cross) toward the ROIs with significantly different Indegree (marked with a dot). The thickness of each link varies according to the value of the connection difference.

### Directional Fronto-Posterior Connectivity Indices

The subsequent aim of this study was to quantify the top-down and bottom-up connectivity within the *theta*, *alpha*, *beta*, and *gamma* frequency bands. We first organized brain regions into 3 distinct categories based on their spatial position: frontal, posterior (parieto-occipital), and temporo-central regions. Notably, we excluded temporo-central regions from further analysis, focusing solely on the frontal (*N*_*f*_ = 22) and posterior (*N*_*p*_ = 18) cortical regions reported in Table 3.

**Table 3.** Categorization of the cortical regions in Frontal and Posterior clusters

Frontal	
ROI	Label
Frontal pole	FP
Rostral anterior cingulate	rAC
Medial orbitofrontal	mOF
Rostral middle frontal	rMF
Pars triangularis	pTR
Pars opercularis	pOP
Pars orbital	pOR
Superior frontal	SF
Caudal anterior cingulate	cAC
Caudal middle frontal	cMF
Lateral orbitofrontal	lOF

Posterior	
Lateral occipital	LO
Cuneus	CU
Pericalcarine	PCL
Lingual gyrus	LG
Superior parietal	SP
Inferior parietal	IP
Precuneus	PCU
Fusiform gyrus	FU
Isthmus	IST

Abbreviation: ROI, regions of interest.

Following this categorization, we pooled together the results obtained on local network metrics (Outdegree and Indegree on individual ROIs), striving to synthesize the information into a unique fronto-posterior and a posterior-frontal connectivity index, for each subject and frequency band. We opted for this approach with the aim of verifying a potential frequency-specific distinction in the feedforward and feedback connectivity within the fronto-parietal regions, and possible anomalies in HSG akin to what has been already observed in schizophrenia patients,^68^ and as predicted by our biobehavioral model.^40^

Our approach initially entailed separate analyses on the Outdegree and Indegree networks, with the aim of distinguishing between fronto-posterior and posterior-frontal connectivity directions.

For Outdegree metrics, we first derived 2 distinct subnetworks: *W*_*out_fp*_ and *W*_*out_pf*_. Specifically, *W*_*out_fp*_ was constructed selecting only the connections exiting from the significant frontal ROIs and directed towards all possible posterior ROIs. Similarly, *W*_*out_pf*_ was obtained by selecting only the connections exiting from the significant posterior ROIs and directed towards all possible frontal ROIs. In order to obtain an average connectivity strength characteristic of the given subnetwork, all the connections within each subnetwork were summed, and the total was then divided by the size of the respective subnetwork, obtaining a single connectivity index for each direction, as outlined below:


$$\begin{array}{l} Out_{FP}=\frac{\sum W_{out\_fp}}{out\_fp};\;Out_{PF}=\frac{\sum W_{out\_pf}}{out\_pf} \end{array}$$

Here, *out_fp* denotes the size of the Outdegree fronto-posterior network (*out_fp* = *N*_*p*_ × *N*_*fo*_, where *N*_*p*_: number of all posterior regions and *N*_*fo*_: number of significant frontal nodes in terms of Outdegree). Similarly, *out_pf* represents the size of the Outdegree parieto-frontal network (*out_pf* = *N*_*f*_ × *N*_*po*_, where *N*_*f*_: number of all frontal regions and *N*_*po*_: number of significant posterior nodes in terms of Outdegree).

Likewise, for the Indegree-based metrics, we first derived 2 distinct subnetworks: *W*_*in_fp*_ and *W*_*in_pf*_. Specifically, *W*_*in_fp*_ was constructed by selecting only the connections exiting from all possible frontal ROIs and directed towards significant posterior ROIs. Similarly, *W*_*in_pf*_ was obtained by selecting only the connections exiting from all possible posterior nodes and directed towards significant frontal ROIs.

Subsequently, the connections within each subnetwork were summed, and the total was then divided by the size of the respective subnetwork, obtaining a single connectivity index for each connectivity direction, as outlined below:


$$\begin{array}{l} In_{FP}=\frac{\sum W_{in\_fp}}{in_{fp}};\;\;\;In_{PF}=\frac{\sum W_{in\_pf}}{in_{pf}} \end{array}$$

Where *in_fp* denotes the size of the Indegree fronto-parietal network (*in_fp* = *N*_*pi*_ × *N*_*f*_, where *N*_*pi*_: number of significant posterior nodes in terms of Indegree and *N*_*f*_: number of all frontal regions). Similarly, *in_pf* represents the size of the Indegree parieto-frontal network (*in_pf* = *N*_*fi*_ × *N*_*p*_, where *N*_*fi*_: number of significant frontal nodes in terms of Indegree and *N*_*p*_: number of all frontal regions).

Finally, for each frequency band, we computed the average of the Outdegree and Indegree metrics to obtain a unique connectivity index for each direction (*CI*_*fp*_: fronto-posterior and *CI*_*pf*_: posterior-frontal).

For this analysis, LMMs were used, considering subjects as random factors. Specifically, we investigated the effects of group (LSG and HSG), frequency band (*theta*, *alpha*, *beta*, and *gamma*), and direction (fronto-posterior and posterior-frontal), as well as their interaction, in predicting the directional connectivity indices. Next, we conducted planned comparisons using 2-tailed Student’s *t*-tests and corrected for multiple comparisons employing the Bonferroni method.

## Results

First, we looked at the *LE* in the 2 experimental groups and across different frequency bands. *LE* represents an index of the segregation tendency of the brain network, where higher values would indicate more efficient communication between the neighboring nodes. Here, we found a main effect of frequency bands (χ^2^(3) = 273.68, *P* < .0001) with an overall higher *LE* for *theta* and *alpha* relative to other frequencies. Likewise, there was a significant main effect of group (χ^2^(1) = 20.05, *P* < .0001), with an overall lower *LE* in the HSG, relative to the LSG. Moreover, a significant interaction was found between frequency bands and groups (χ^2^(3) = 28.45, *P* < .0001), meaning that the difference in *LE* between the 2 groups varies across frequencies. Specifically, Bonferroni-corrected planned contrasts (*P* < .012) revealed that *LE* is significantly lower in the HSG versus LSG across *theta* and *alpha* frequency (all *t*s(107) > 3.760, all *P*s < .0004), but not in *beta* and *gamma* (all *t*s(107) < 1.426, all *P*s > .100). Taken together, these analyses would indicate that HSG has a substantial decrease in information segregation tendency, which would translate into less efficient brain organization, compared to LSG, especially evident across *theta* and *alpha* frequency.

We also looked at the *GE*, which represents the integration tendency of the network, where higher values of *GE* would indicate more efficient brain network organization. Again, here we observed the main effect of frequency bands (χ^2^(3) = 304.38, *P* < .0001), with greater network efficiency in the *theta* and *alpha* range, compared to *beta* and *gamma* frequencies. Moreover, we found a significant main effect of group (χ^2^(1) = 18.062, *P* < .0001), with an overall reduced *GE* for the HSG, reflecting a lower integration tendency in these participants. Likewise, a significant interaction was found between frequency bands and group (χ^2^(3) = 30.87, *P* < .0001). The planned contrasts revealed that the *GE* is significantly lower in the HSG (*t*-test statistics, Bonferroni-corrected *P*-value) across *theta*, *alpha*, and *beta* frequencies (all *t*s(107) > 2.541, all *P*s < .012), but not in the *gamma* frequency (*t*(107) = 1.103, *P* = .271) (see Figure 1).

Taken together, these results show that high-schizotypy individuals have a lower *LE* in the *theta* and *alpha* band, and a reduced *GE* across *theta*, *alpha*, and *beta* frequencies, with both lower *LE* and *GE* marking a less efficient network organization.

To strengthen the robustness of our findings, we also performed the same analysis by using the alternative connectivity measures, thus offering a complementary perspective to GC.^69^ Similar to the GC, the results revealed that both the *LE* and *GE* are significantly lower in the HSG across *theta* and *alpha* frequencies, while the differences are less pronounced across higher *beta* and *gamma* frequencies (for more details, see Supplementary Figure S3).

Figure 1 presents bar plots (mean ± SEM) illustrating resting-state Global Network topology indices for both LSG and HSG across the *theta*, *alpha*, *beta*, and *gamma* frequency bands. Specifically, Figure 1A shows the *LE*, and Figure 1B shows the *GE*.

Figure 2 shows the connection differences exiting from the brain nodes whose Outdegree is significantly different between HSG and LSG in *theta* (Figure 2A), *alpha* (Figure 2B), *beta* (Figure 2C), and *gamma* (Figure 2D) frequency bands. Lines denote higher connectivity for the LSG. It is worth noting that in this analysis, we employed sparse connectivity matrices. This means that only connections displaying a significant difference between the 2 groups (uncorrected *P*-value) were taken into consideration.

In terms of Outdegree, 5 nodes have been identified for the *theta* band (pTR l, pOR, ST l, LG l, and LG r), 2 for the *alpha* band (cAC l and cAC r), and 2 for the *beta* band (pTR l and TP l) as significantly different between the 2 groups (Bonferroni-corrected *P*-value). Interestingly, Figure 2 illustrates that, regardless of the frequency band, the Outdegree, and thus the connections exiting from the identified nodes, are always higher in the case of low-schizotypy subjects and never for high-schizotypy subjects. Figure 3 shows the main connection differences entering into the brain nodes whose Indegree is significantly different between HSG and LSG in *theta* (Figure 3A), *alpha* (Figure 3B), *beta* (Figure 3C), and *gamma* (Figure 3D) frequency bands. Lines denote higher connectivity for the LSG. In terms of Indegree, 1 node has been identified for the *theta* band (PCL r), 2 for the *alpha* band (PH l and EN l), 1 for the *beta* band (cAC r), and 1 for the *gamma* band (cAC r) as significantly different between the 2 groups (Bonferroni-corrected *P*-value). Again, Figure 3 illustrates that, regardless of the frequency band, the Indegree, and thus the connections entering into the identified nodes, are always higher in the case of low-schizotypy subjects and never for high-schizotypy subjects. Overall, Figures 2 and 3 show a systemic reduction in functional connectivity in the HSG. Moreover, this reduction mainly involves the areas that are part of the default mode network (such as temporal pole: TP, entorhinal cortex: EN, parahippocampus: PH, and posterior cingulate cortex: PCC), auditory and language processing network (such as pars triangularis: pTR, pars opercularis: pOP, and superior temporal lobe: ST), and areas involved in social and emotional regulation (such as caudal anterior cingulate: cAC and banks of superior temporal sulcus: BK).

**Figure 4.**
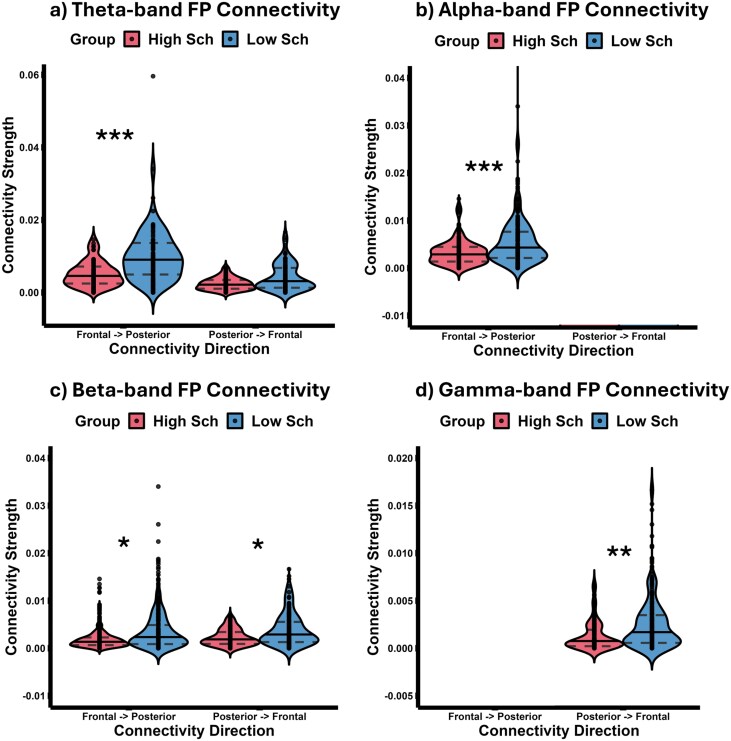
Violin plots representing the distribution of fronto-posterior and posterior-frontal connectivity indices for Low-Schizotypy Group and High-Schizotypy Group and for *theta* (A), *alpha* (B), *beta* (C), and *gamma* (D) frequency bands. Data are presented as median (full line) ± 1 quartile (dashed line).

Figure 4 displays violin plots illustrating the central tendency and distribution of fronto-posterior and posterior-frontal connectivity indices between individuals with high and low schizotypy across the *theta* (Figure 4A), *alpha* (Figure 4B), *beta* (Figure 4C), and *gamma* (Figure 4D) frequency bands. It should be noted that the posterior-frontal connectivity index is absent in *alpha* band, while the fronto-posterior connectivity index is missing in *gamma* band. We found a significant main effect of group (χ^2^(4) = 187.90, *P* < .0001), meaning that the fronto-posterior and the posterior-frontal indices of connectivity are significantly lower in the HSG for all frequency bands. Moreover, we highlight a significant main effect of frequency (χ^2^(1) = 152.47, *P* < .0001), with faster (*beta* and *gamma)* frequencies having an overall lower long-range connectivity strength. This finding is not surprising, as it goes well in hand with the common finding of lower oscillatory frequencies (such as *theta* and *alpha*) being more prominent in long-range between-areal synchronization.^70,71^ Crucially, we also observed a significant 3-way interaction between connectivity direction, frequency, and group (χ^2^(8) = 72.33, *P* < .0001). In other words, the differences between the 2 groups follow a frequency- and direction-dependent trend. Specifically, as revealed by Bonferroni-corrected planned contrasts (*P* < .006), in slower frequency bands, such as *theta* and *alpha*, there is a marked reduction in fronto-posterior (top-down) connectivity in the HSG relative to the LSG (*theta*: *M*_low_ = 0.010, *M*_high_ = 0.005, *t*(213) = 5.977, *P* < .001; *alpha*: *M*_low_ = 0.006, *M*_high_ = 0.003, *t*(213) = 3.577, *P* < .001), but not in the posterior-frontal (bottom-up) connectivity (*theta*: *M*_low_ = 0.005, *M*_high_ = 0.002, *t*(213) = 2.327, *P* = .021). On the other hand, in faster frequency bands such as *beta* and *gamma*, we observe a gradual decrease in posterior-frontal (bottom-up) connectivity in the HSG relative to the LSG, where in the *beta* band, we can observe both top-down (*M*_low_ = 0.003, *M*_high_ = 0.002, *t*(213) = 3.168, *P* = .002) and bottom-up (*M*_low_ = 0.004, *M*_high_ = 0.002, *t*(213) = 3.215, *P* = .002) decrease in connectivity, while in faster *gamma* band, the differences became evident solely in the feedforward direction (*M*_low_ = 0.002, *M*_high_ = 0.001, *t*(213) = 3.752, *P* < .001).

## Discussion

Synchronous neural oscillations play a central role in brain functioning, underpinning the fundamental mechanisms responsible for communication within multiple large-scale brain networks. These brain rhythms serve as key drivers of functional connectivity, providing the framework that allows brain regions to work in unison and share information efficiently. The investigation of these neural oscillations, their interconnections, and their role in brain function has significantly contributed to our understanding of how the brain processes information, adapts to dynamic environmental demands, and contributes to the development of psychopathological conditions. Therefore, oscillatory coupling between different brain areas during resting state has been identified as a core process of the coordination and integration of signal transmission and information processing between core cortical regions or brain networks.^72,73^ Consequently, aberrant connectivity in schizophrenia could indicate dysfunctional between-areal communication, possibly explaining some of their related symptoms.

According to the notion that schizophrenia disorder represents a disconnection syndrome,^4–6^ here we investigate whether the same alterations of the functional connectivity could be found in the healthy, nonclinical population showing high schizotypal traits. To this aim, we used resting-state EEG activity combined with directional functional connectivity and graph theory metrics, to compare the spectral brain network patterns of individuals assigned to HSG and LSG. Directional functional connectivity was extracted using spectral GC. This choice is supported by prior research comparing various functional connectivity estimators with Neural Mass Models, which found GC to be the most reliable method.^74^

First, we found an overall lower *LE* in HSG, especially pronounced across *theta* and *alpha* frequencies, along with a lower *GE* in *theta*, *alpha*, and *beta* frequency bands, demonstrating a less efficient network organization in this group. Second, the directed degree centrality analysis showed that all incoming and outgoing connections are stronger in the LSG than HSG, revealing a systematic reduction of brain connectivity in the HSG. Third, feedback connections differences (from frontal to posterior brain areas) are mostly pronounced in the lower (*theta* and *alpha*) frequency range, while feedforward connection differences (from posterior to frontal brain areas) are mostly found across higher (*beta* and *gamma*) frequencies, demonstrating frequency-specific differences in the top-down and bottom-up connectivity pathways. Taken together, the current results confirm the existence of a compromised functional brain connectivity in individuals with high schizotypy, indicating that the network alterations observed in schizophrenia are already present before the onset of psychosis, and may represent an early indicator of predisposition and risk for psychosis development.

The use of graph theory metrics allowed us to assess differences in the efficiency of brain network architecture across the schizotypy dimension. Efficient networks are described by the combination of high *LE* (indicating dense local clustering among neighboring nodes) and a high *GE* (reflecting relatively few long transmission pathways). This topology has been proposed as an optimal organization for cortical information exchange, since it can support both segregated/specialized and distributed/integrated information processing. Additionally, these optimal networks are also cost-effective, as they tend to minimize wire costs while sustaining high dynamical complexity.^75–77^ Here, we observed that HSG individuals exhibit a weaker *LE* together with reduced *GE*, indicating a disorganization of their functional network’s efficiency compared to LSG individuals. Similar findings have also been reported in functional MRI studies of individuals with schizophrenia, showing alterations in functional networks characterized by a reduced *GE* and *LE*.^12,14^

Furthermore, consistent findings suggest that neural oscillations at both low and high frequencies play a crucial role in the pathophysiology of schizophrenia, potentially underlying the difficulties in generating coherent cognition and behavior.^78^ For instance, studies have shown that irregularities in the synchrony of *gamma*-band oscillations play a crucial role in cognitive deficits.^79^ Recent research has highlighted increased resting-state *theta*-band connections between the posterior cingulate cortex, cuneus, and precuneus, hindering the timely initiation of cognitive tasks in first-episode schizophrenia patients.^80^ Another study on schizophrenia revealed disrupted functional connectivity in the *alpha*, *beta*, and *gamma* bands during resting state.^15^ Our findings align with this body of research, demonstrating that the impairment of frequency-specific brain network efficiency is already present in healthy individuals with an elevated risk of developing psychosis.

Moreover, individuals with high schizotypy scores exhibited a lower nodes centrality and a systematic decrease in connectivity, reflecting disconnection between brain regions. In sum, frontal hubs in HSG show a lower capacity to drive information towards posterior brain areas in lower frequencies. On the other hand, posterior brain areas in HSG show a lower capacity to drive information towards frontal areas in the higher frequencies. These results align with aberrant functional brain connectivity found in schizophrenia patients. For instance, a study focusing on adolescent-onset schizophrenia identified alterations in the right inferior frontal lobe, fusiform gyrus, and thalamus, implicating their role in symptom severity.^81^ Another study revealed distinct temporal connectivity patterns in first-episode schizophrenia patients, with significant differences in regions such as the fusiform gyrus, cingulate cortex, and superior parietal gyrus between treatment responders and nonresponders.^82^ These areas are involved in a variety of functions such as language processing, memory, visual perception, and executive functions, indicating a more general connectivity deficit across different brain circuits in HSG. In line with this conclusion, we also found that the areas that demonstrate a reduced functional connectivity are part of the default mode network (posterior cingulate cortex, temporal pole, entorhinal cortex, and parahippocampus), a network mostly activated during rest, as well as the areas involved in language processing and social and emotional regulation (pars opercularis, pars triangularis, superior temporal lobe, and anterior cingulate). This fits well both the interpersonal (social anxiety, no close friends, constricted affect, paranoid ideation) and disorganized features (odd behavior, odd speech) of HSG. Moreover, both bottom-up (feedforward) and top-down (feedback) connectivity is altered, though across different frequency bands: while the differences in the connections of the fronto-posterior direction are mostly visible in the lower (*theta* and *alpha*) frequency bands, the differences across the higher frequency bands between the 2 groups are mostly present in the opposite, postero-frontal direction. Previous research has linked slower and higher oscillatory activity to feedback and feedforward mechanisms, respectively.^71,83,84^ Therefore, the reduced connectivity in HSG compared to LSG seems to follow this dominant frequency dependency. Although previous theoretical models have linked schizophrenia dimension to a specific alteration of the feedback mechanism,^40^ our results indicate that feedforward connections might similarly be altered, speaking in favor of an overall functional dysconnectivity in schizotypy, not limited to a distinctive information flow.

From the analysis conducted across LSG and HSG (see Figures 2–4), we could notice a clear difference between the LSG and HSG in the distribution of the graph theory indices, with LSG showing more pronounced within-group variability (ie, long-tailed distributions for the LSG). This higher interindividual differences in LSG could be explained by other factors not accounted for here. In other words, while in HSG, we have a specific personality conglomerate which seems to determine an alteration of brain organization, LSG group could still highly vary on different possible determinants, such as variability in other personality^85,86^ or cognitive features,^87^ which could further explain this variance in brain network organization.

On a similar note, we cannot exclude the possibility that a similar pattern of brain connectivity changes is not present in other traits and/or patient groups, which often co-occur with schizotypy. For instance, the negative subscale of SPQ highly correlates with some autistic features,^88^ while symptoms of depression and anxiety are associated with the positive-symptom dimension of schizotypy,^89^ consistent with studies of schizophrenic patients.^90^ Therefore, future research should control these factors by administrating additional questionnaires aimed at estimating autistic traits, or prevalence of anxiety and depressive symptoms.

Finally, the question remains if this altered resting-state functional connectivity persists during perceptual and cognitive processing,^91–93^ as it happens to be the case in schizophrenia patients. Although altered perception and cognition have already been linked to schizotypy,^91,94,95^ it is up to future research to link these behavioral changes to possible alterations of the functional brain circuits during task.

In sum, the current study employed graph theory metrics to reveal alterations in frequency-specific functional brain networks in individuals with high schizotypy. Our findings indicate an overall less efficient organization of functional networks, as highlighted by lower *LE* and *GE* in these individuals. Moreover, we observed a lower nodes centrality and a systematic reduction in connectivity in high schizotypy, driven by frequency-dependent bottom-up and top-down processing pathways. Taken together, these results provide further support for the dimensional model of schizophrenia, suggesting that alterations in functional brain connectivity are already present in healthy individuals with higher schizotypal traits, and hence at higher risk of developing psychosis.

## Supplementary Material

Supplementary material is available at https://academic.oup.com/schizophreniabulletin.

sbaf004_suppl_Supplementary_Figures
