# Supplementary material for: Aberrant Functional Connectivity and Brain Network Organization in High-Schizotypy Individuals: An Electroencephalography Study
Source: Schizophr Bull. 2025 Feb 4;51(5):1266–81. doi: 10.1093/schbul/sbaf004 (PMC12414563; doi:10.1093/schbul/sbaf004)
Supplement: sbaf004_suppl_Supplementary_Figures [file sbaf004_suppl_supplementary_figures.docx]

**Supplementary Materials**

***Demographics***


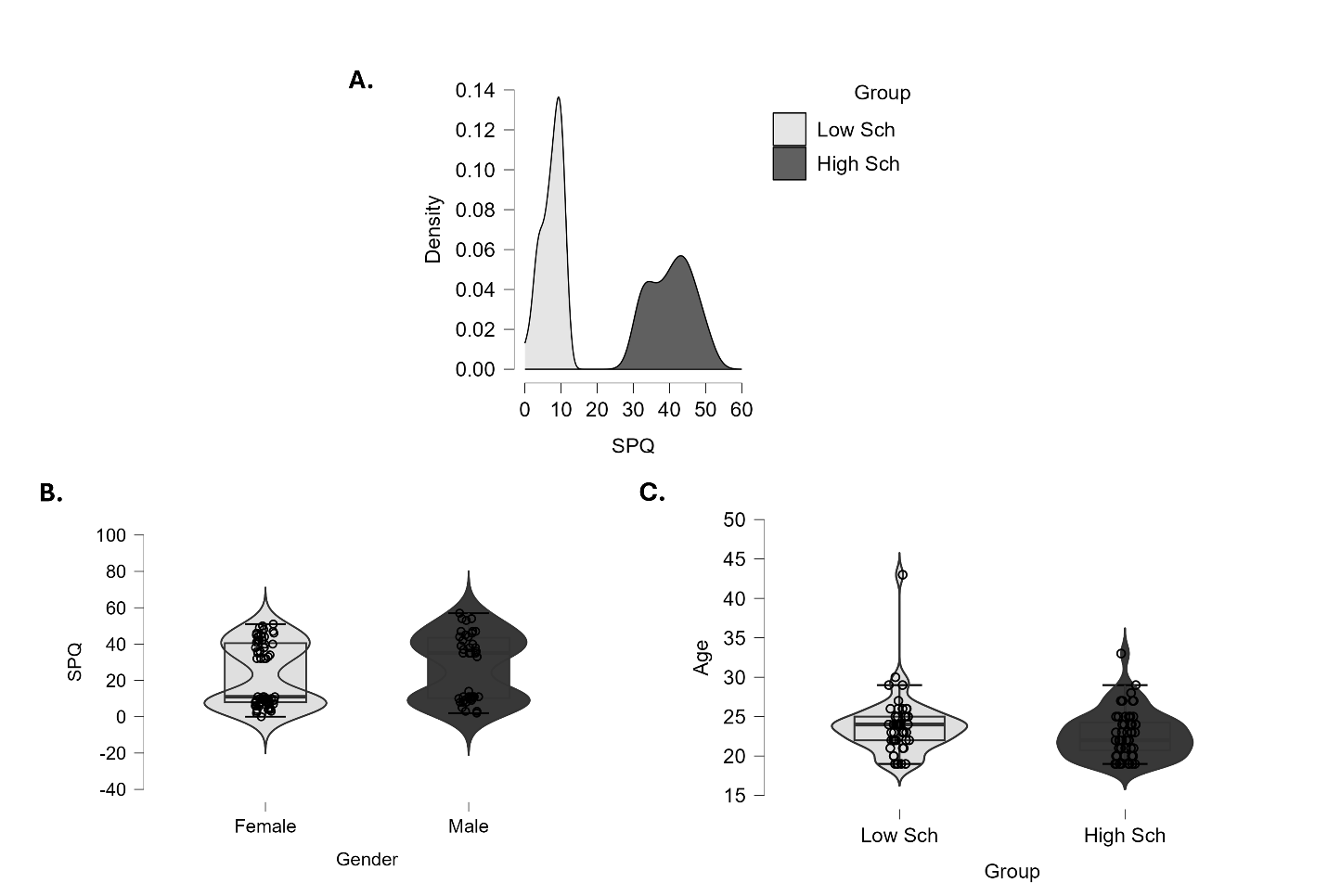


**Figure S1.** Intragroup differences. A. Schizotypy scores distribution: schizotypy as a trait is not normally distributed in the general population: rather, it is highly skewed to towards one end, with a higher number of individuals showing low (compared to high) schizotypy scores^e.g.,1–3^. We can see this from the SPQ distribution for Low (LSG, light gray) and High Schizotypy group (HSG, dark gray) where HSG has a much wider score range B. Schizotypy scores and gender: There were no significant gender differences between the groups (χ2 = 0.506, p = .477). C. Schizotypy scores and age: There were no significant age differences between the two groups (Mlow = 23.784 ± 3.743, Mhigh = 22.750 ± 3.002, t = 1.548, p = .125).

***Granger Causality: Complete vs Sparse***

1. ***Adjacency Matrix Sparsification***

In the following section, we compare the results obtained using the complete connectivity matrices with their sparsification derived from statistical analysis. Figures S2.i: a)-d) display the adjacency matrices for the theta, alpha, beta, and gamma frequency bands.

**a)**


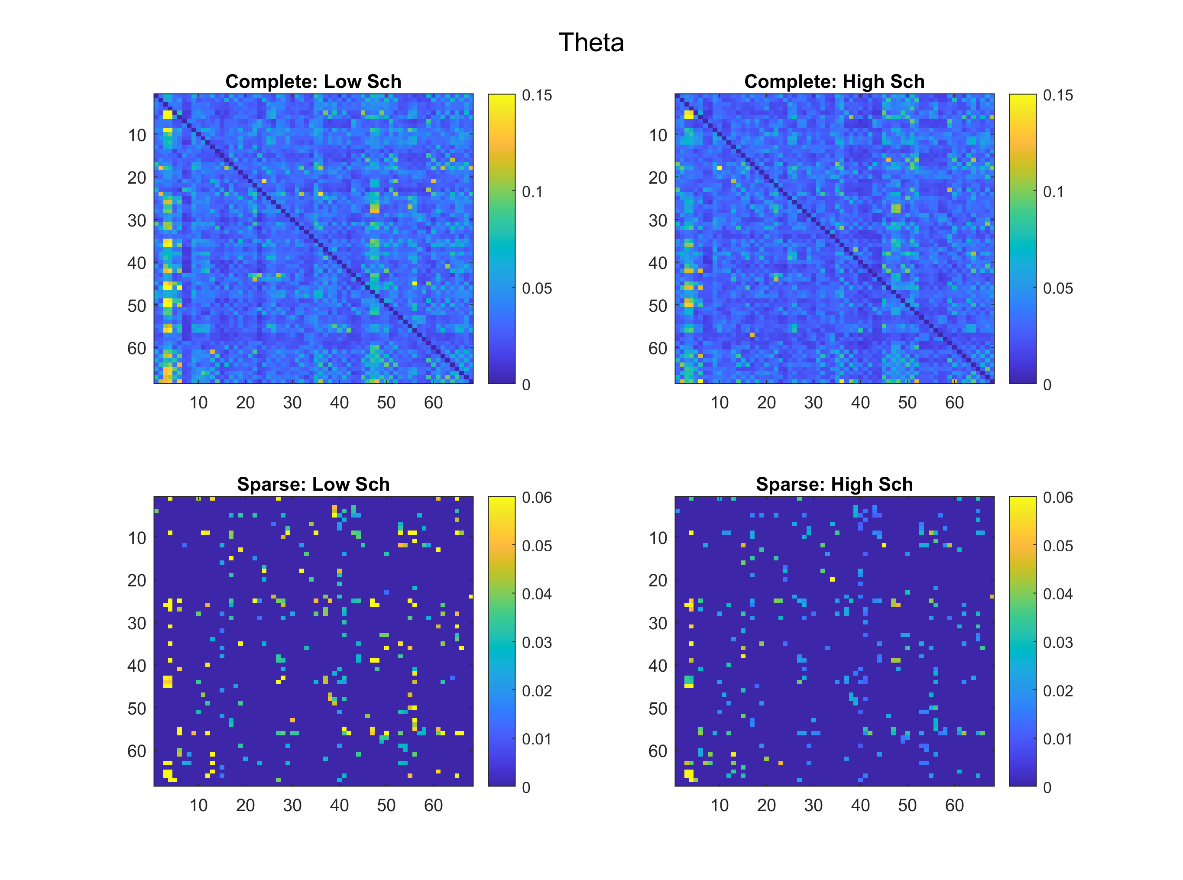


**b)**


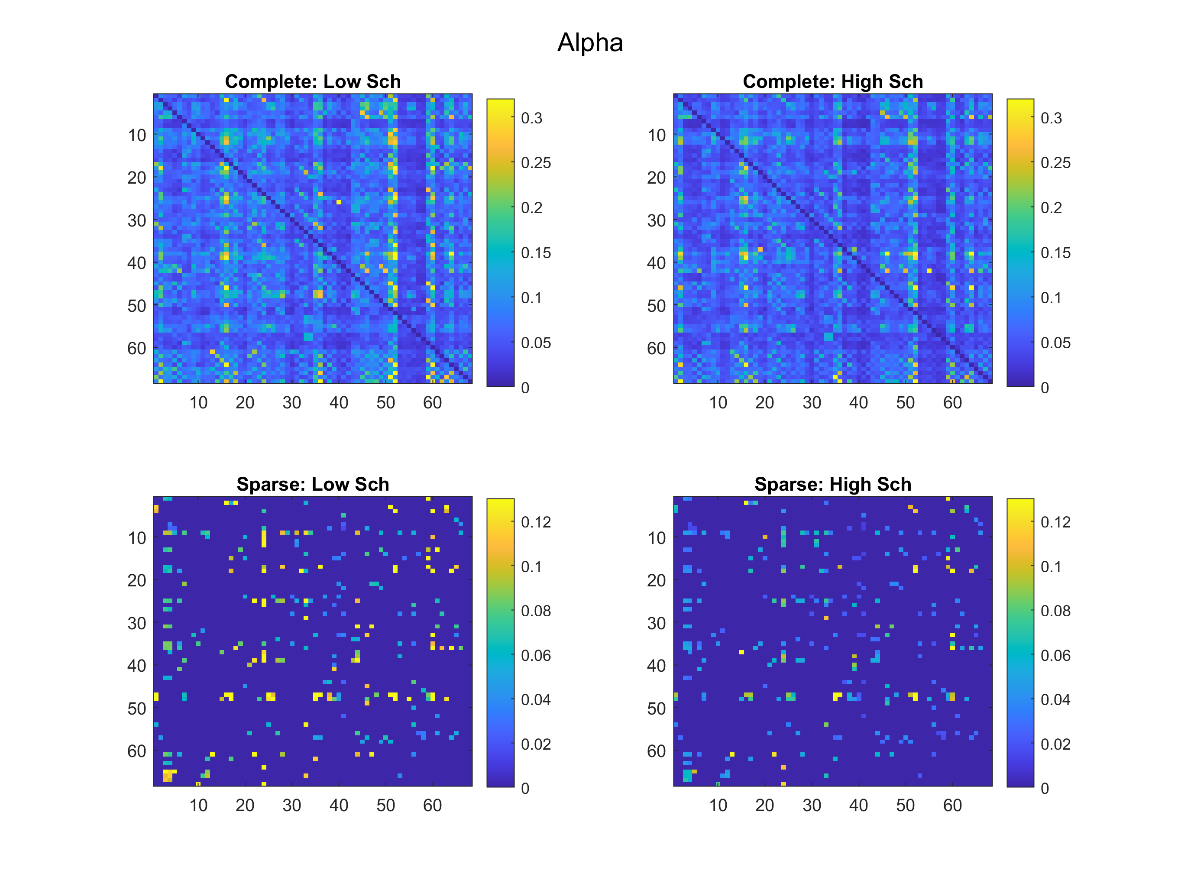


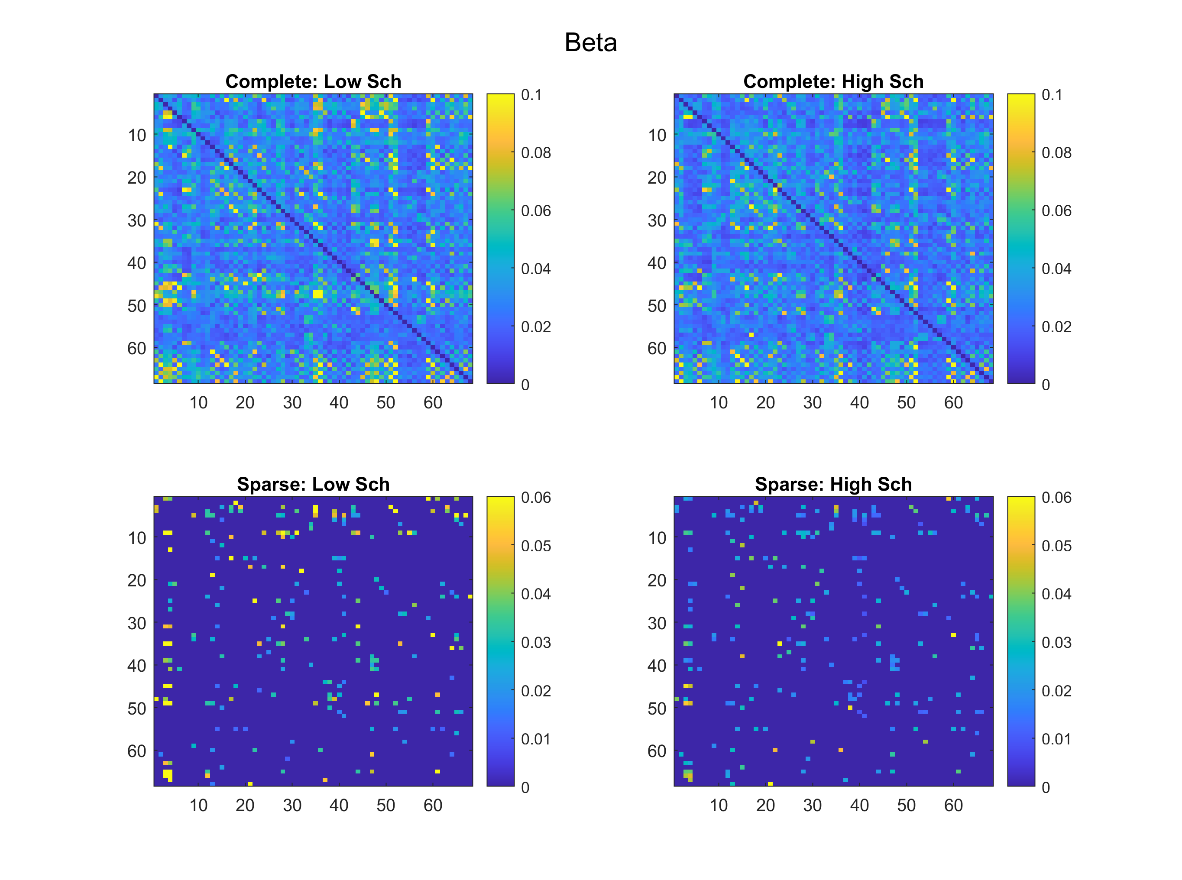


**c)**


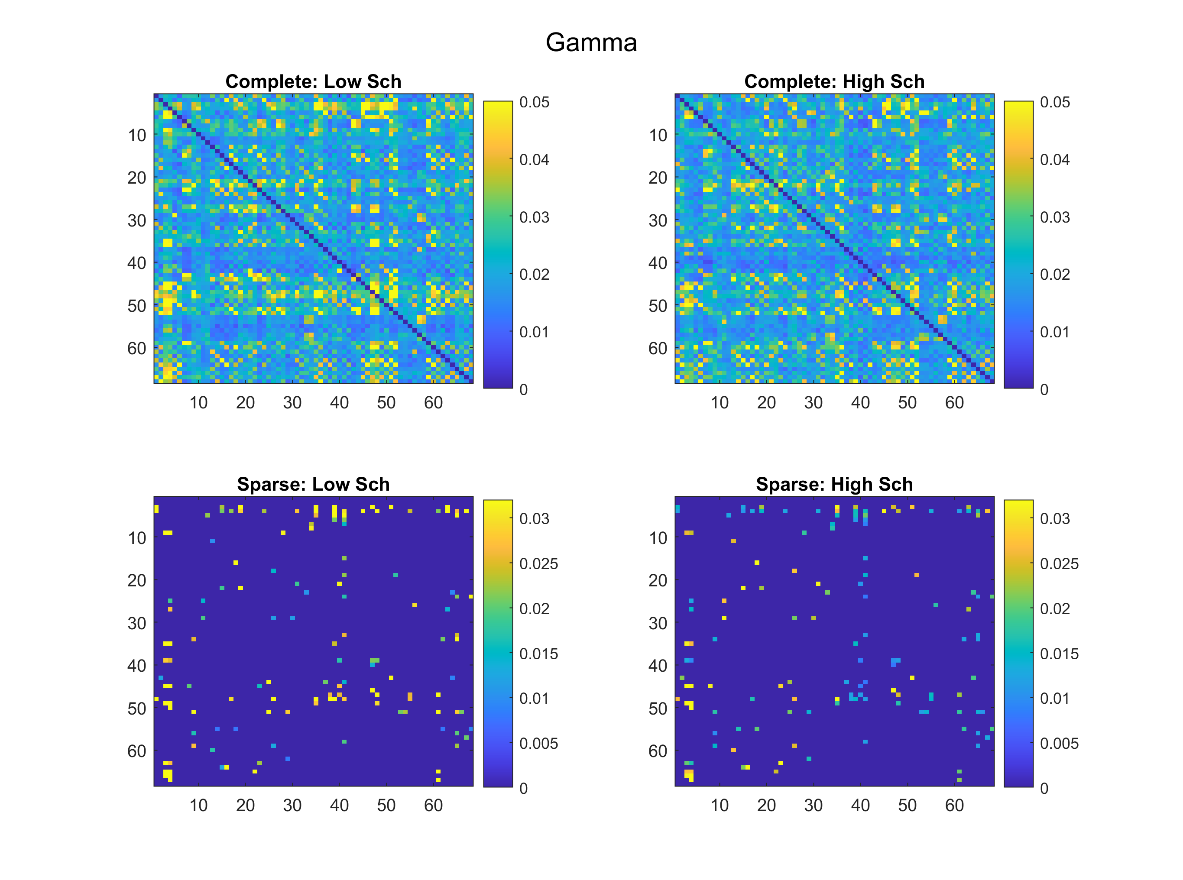


**d)**

**Figure S2.i**. Granger Causality Complete and Sparse Connectivity: Adjacency Matrices. Subfigures a), b), c), and d) display the complete (top panels) and sparse (bottom panels) adjacency matrices for the Low (left) and High (right) Schizotypy Groups across different frequency bands (theta, alpha, beta, gamma).

1. ***Global Indices: Complete vs Sparse***

This section compares global connectivity indices, specifically local and global efficiency, in the case of complete and sparse connectivity matrices. The aim is to assess how these indices differ when using the complete matrices versus the statistically reduced, sparse versions. By doing so, we can evaluate the impact of sparsification on the overall network's ability to efficiently process and transfer information. Figure S2.ii shows that the trend (Low>High) between complete and sparse matrices is maintained for both measures of Local and Global Efficiency, and that in the sparse case the difference between the groups is even more pronounced.

**Complete Connectivity Matrices**

**a)**


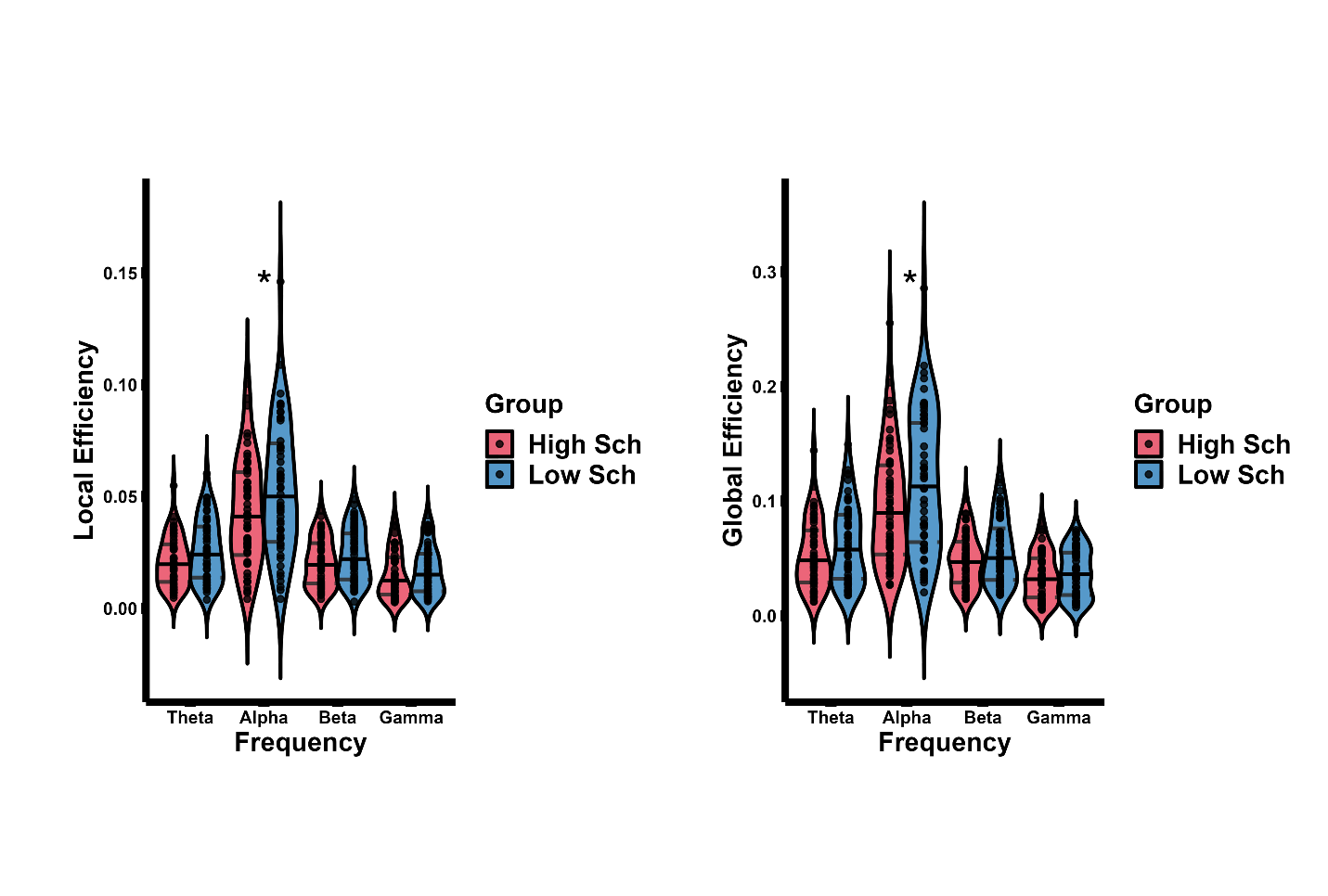


**Sparse Connectivity Matrices**

**b)**


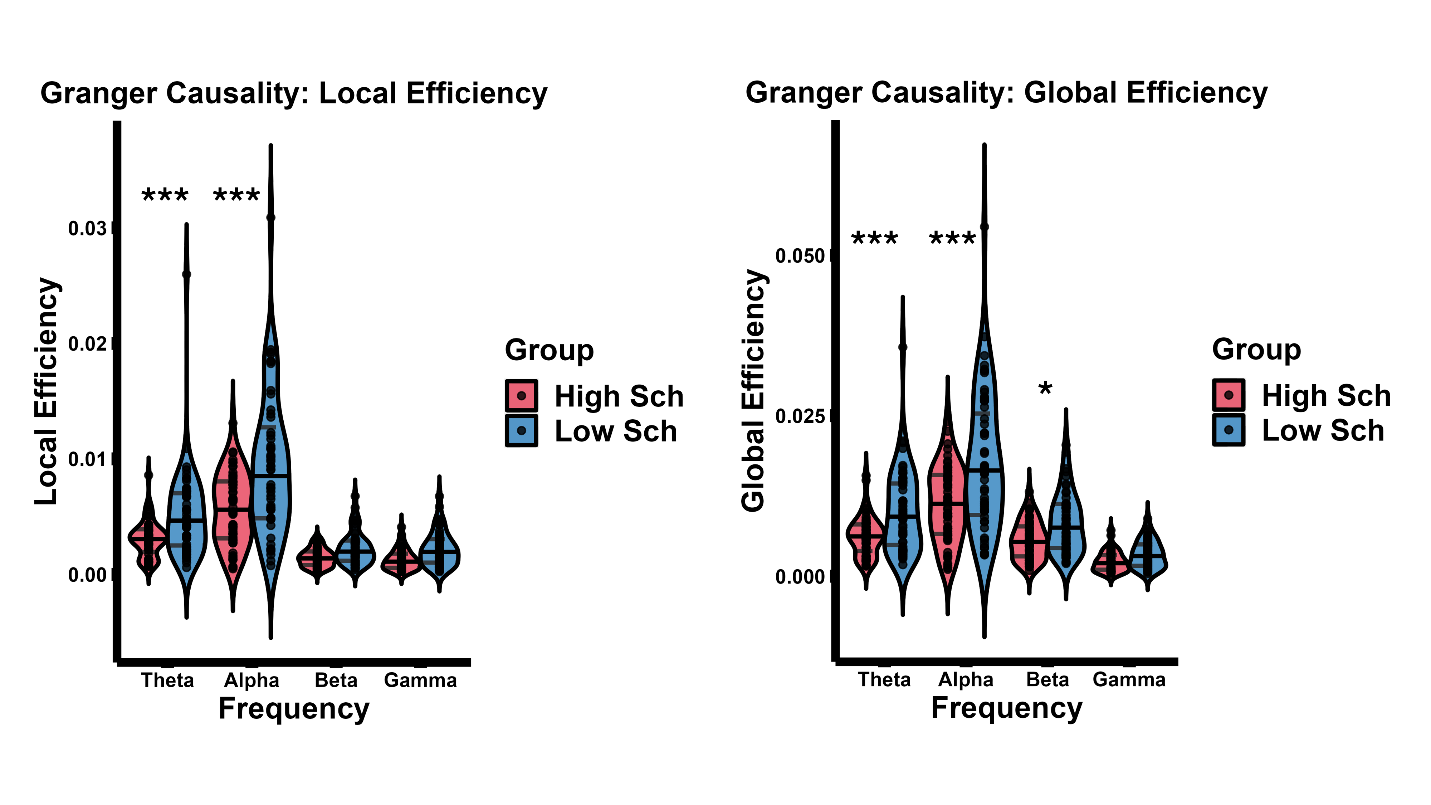


**Figure S2.ii**. Granger Causality Complete and Sparse Connectivity: Global Indices. Violin plots representing the Global Network topology indices for Low (blue) and High (red) Schizotypy groups and for theta, alpha, beta and gamma frequency bands. Panel a) shows the Local (left) and Global (right) Efficiency for the complete matrices. Similar to the results on the sparse matrices, for both LE and GE, there was a significant interaction between the frequency band and group (LE: χ2 (3)=8.545,p=.036; GE: χ2 (3)=9.593,p=.022), with a lower global and local efficiency in the alpha band in HSG compared to LSG (Bonferroni corrected significance threshold: p<0.0125all ts> 3.016, p<0.003), which was not evident in other frequency bands (Bonferroni corrected significance threshold: p<0.0125; all ts<1.499, all ps>.135). Panel b) shows the Local (left) and Global (right) Efficiency for the sparse matrices (as in the main text). Data are presented as median (full line) ± 1 quartile (dashed line).

1. ***Granger Causality Thresholded Complete Network***

In this section, a thresholded version of Low and High Schizotypy connectivity network is presented for each frequency band (theta, alpha, beta and gamma). Specifically, the threshold for each band is set to 30% of the maximum absolute difference between the Low Schizotypy and High Schizotypy groups. This means that only connectivity differences exceeding this threshold are represented in the network. By applying this threshold, we aim to emphasize the main connectivity differences between the two groups across the theta, alpha, beta, and gamma bands, providing a clearer visualization of key network changes. Figure S2.iii depicts the difference in connectivity between the two groups, with the blue arrows representing the greater connections in the Low Schizotypy group and the red arrows representing the greater connections in the High Schizotypy group.


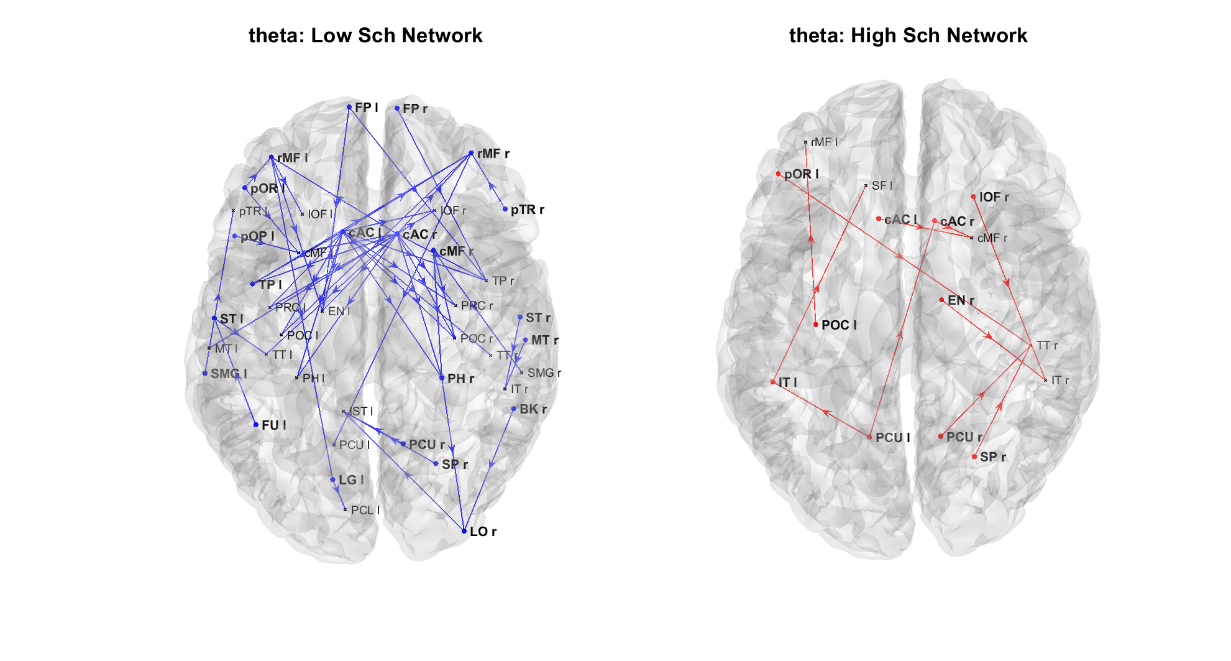


**b)**

**a)**

**b)**

**a)**

**c)**


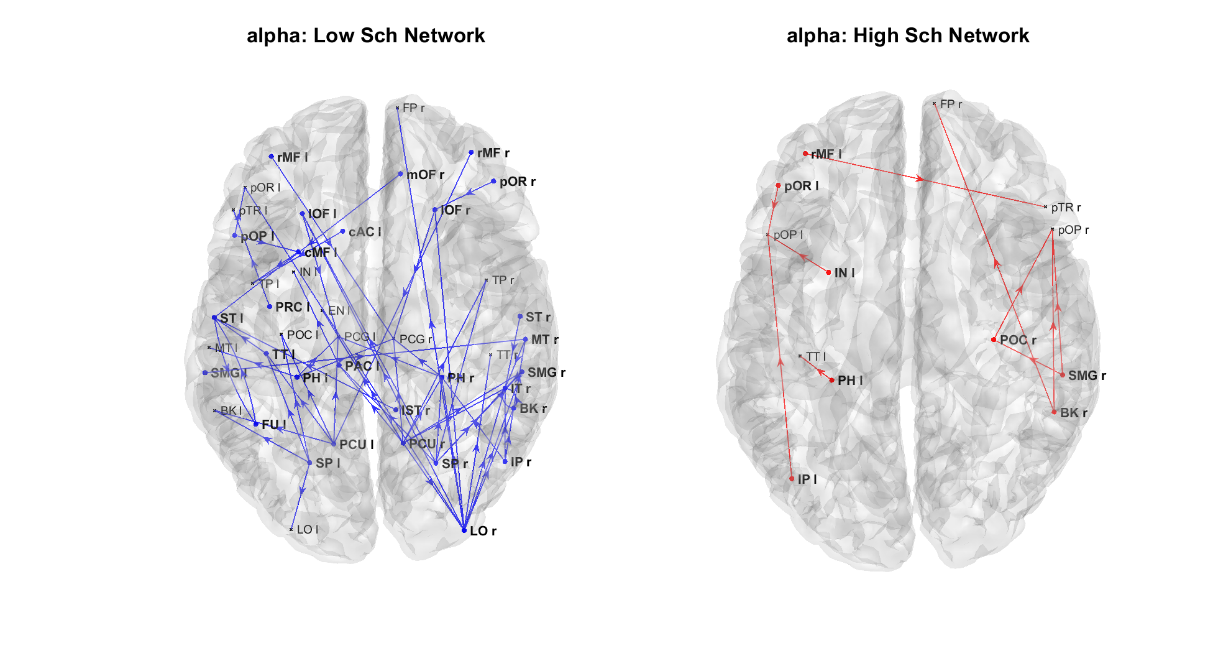


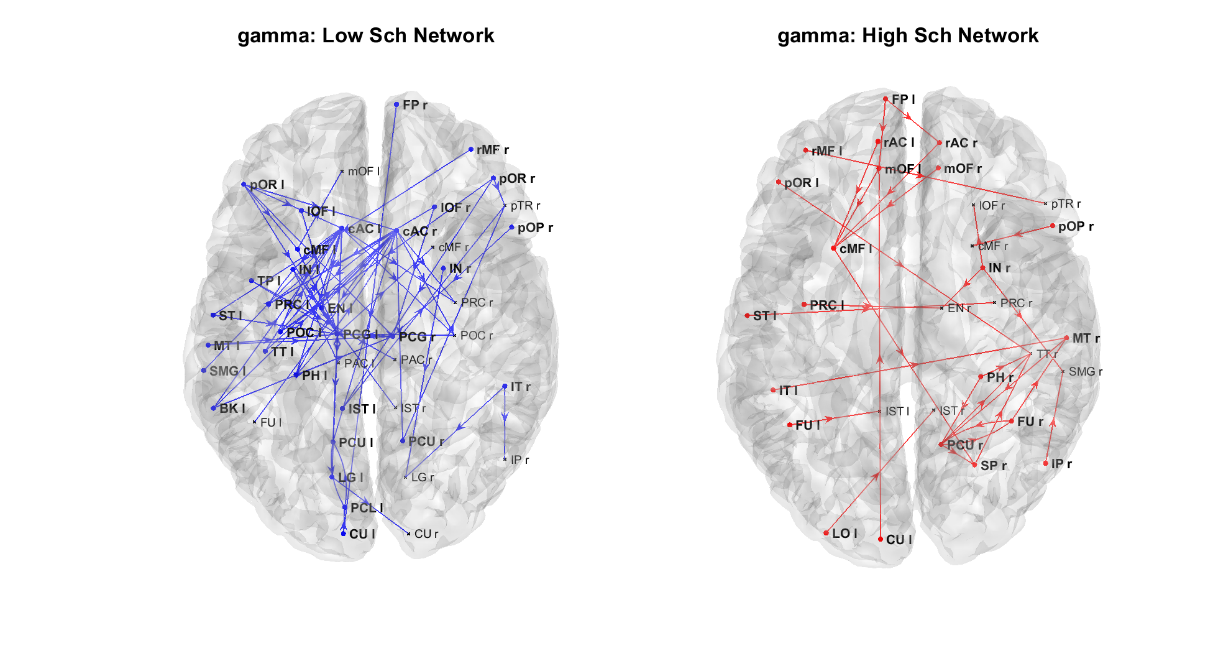

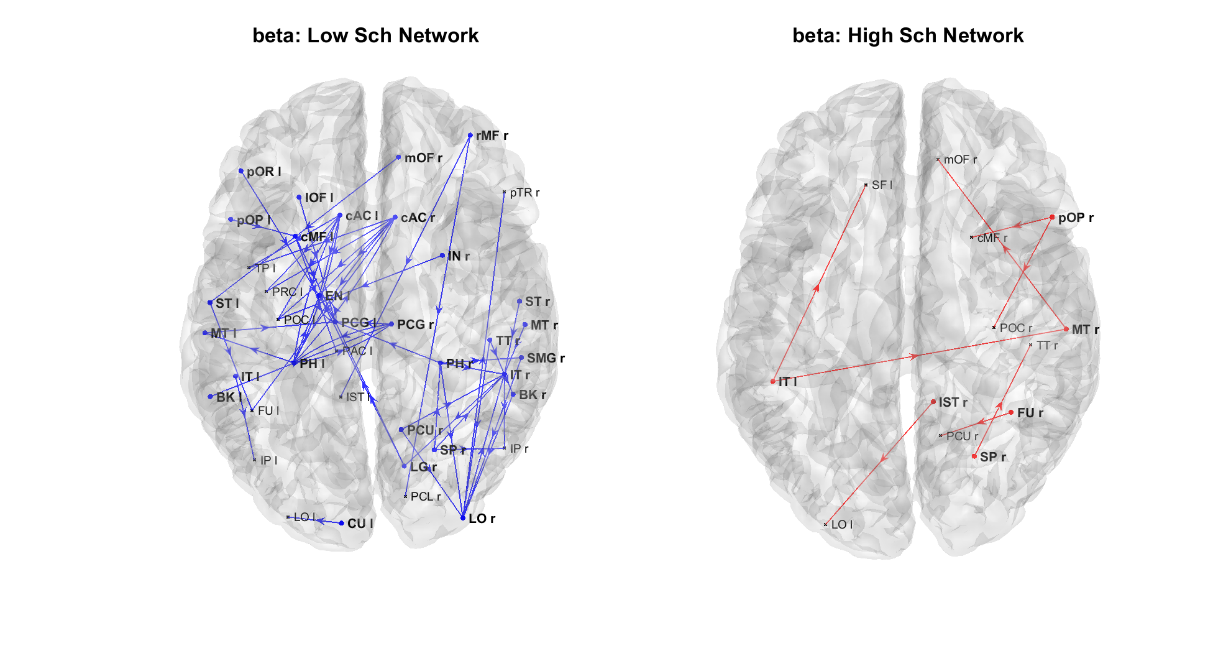


**d)**

**Figure S2iii**. Granger Causality Thresholded Complete Network. This figure shows, for each frequency band, the brain network resulting from the connectivity difference between Low and High Schizotypy groups, considering a threshold of 30% of the maximum absolute connectivity difference. Only connections exceeding this threshold are displayed. Subfigures show differences across frequency bands: (a) theta, (b) alpha, (c) beta, and (d) gamma. Blue lines and nodes indicate stronger connectivity in the Low Schizotypy group, while red lines and nodes highlight stronger connectivity in the High Schizotypy group.

***Non-directional Connectivity: Weighted Phase Lagged Index***

We replicated the analyses related to global network indices, using the weighted Phase Lag Index (wPLI) estimator as a non-directional metric of functional connectivity. The wPLI measures the phase difference between two signals, providing insights into the consistency of phase differences while mitigating the influence of volume conduction artifacts. We computed the degree measure of centrality on sparse connectivity matrices (obtained after statistic thresholding) to determine the non-directional network properties of the ROIs, which confirmed a generally stronger network efficiency in the Low Schizotypy group (LE: $\chi^{2}\left( 1 \right)=32.57, p<.0001$; GE: $\chi^{2}\left( 3 \right)=38.05, p<.0001$). Likewise, a significant interaction was found between frequency bands and group for both GE and LE (LE: $\chi^{2}$ (3)=31.99, p< .0001; GE: $\chi^{2}$ (3)=18.80, p = .0003 ). Similar to the Granger Causality, the planned contrasts revealed that both the LE and GE is significantly lower in the HSG (t-test statistics, Bonferroni corrected p-value) across theta and alpha frequencies (all ts(107) > 2.832, all ps<0.005), while the differences are less pronounced across higher beta and gamma frequencies (all ts(107) < 2.304, all ps>.022).

This additional analysis strengthens the robustness of our findings by offering a complementary perspective to Granger Causality, thereby enhancing the comprehensiveness of our study.

**a)**


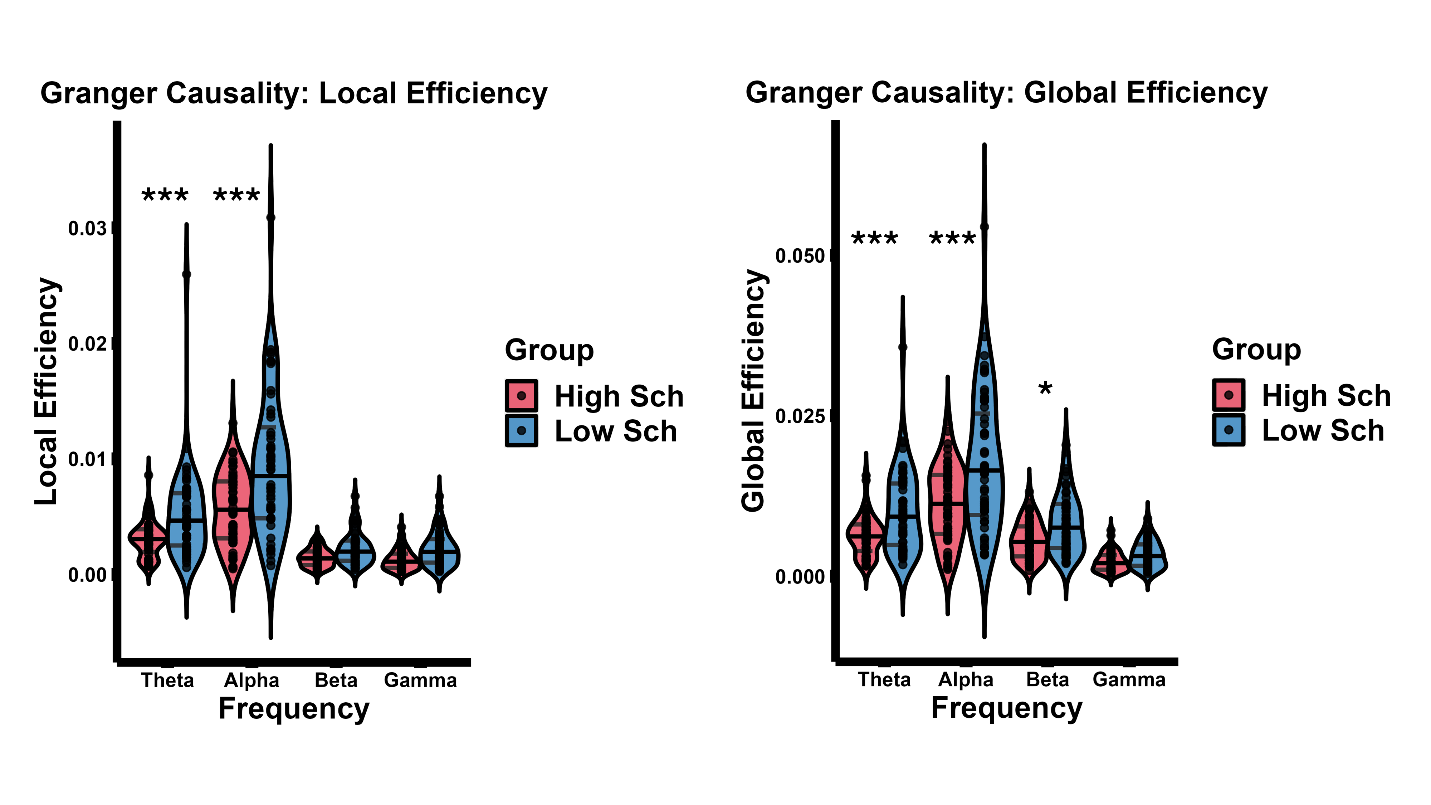


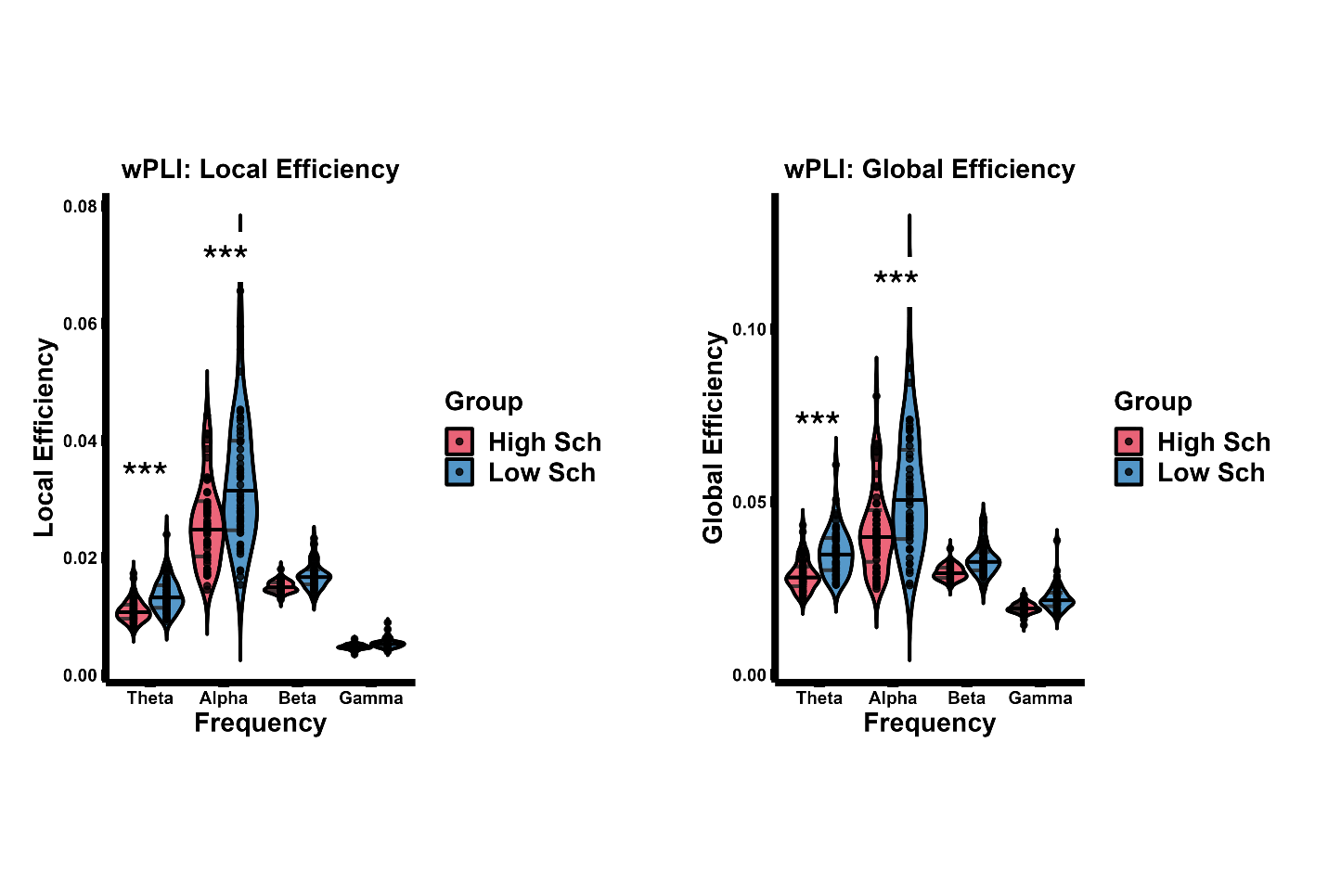


**b)**

**Figure S3.** Granger Causality and Weighted Phase Lag Index Comparison: Global Indices. Violin plots representing the Global Network topology indices for Low (blue) and High (red) Schizotypy groups and for theta, alpha, beta and gamma frequency bands. Panel a) shows the Local (left) and Global (right) Efficiency for the sparse matrices obtained via the Granger Causality estimator. Panel b) shows the Local (left) and Global (right) Efficiency for the sparse matrices obtained via the weighted Phase Lag Index estimator. Similar to the results on the Granger Causality, for both LE and GE, there was a significant interaction between the frequency band and group (LE: χ2 (3)=31.993,p<.0001; GE: χ2 (3)=9.593,p=.022), with a lower global and local efficiency in the theta and alpha band in HSG compared to LSG (Bonferroni corrected significance threshold: p<0.0125: all ts> 2.832, p<0.005), which was not evident in other frequency bands (Bonferroni corrected significance threshold: p<0.0125: all ts<2.306, all ps>.022). Data are presented as median (fullline) ± 1 quartile (dashed line).

**References**

1. Hurst RM, Nelson-Gray RO, Mitchell JT, Kwapil TR. The Relationship of Asperger’s Characteristics and Schizotypal Personality Traits in a Non-clinical Adult Sample. *J Autism Dev Disord*. 2007;37(9):1711-1720. doi:10.1007/s10803-006-0302-z

2. Knolle F, Ronan L, Murray GK. The impact of the COVID-19 pandemic on mental health in the general population: a comparison between Germany and the UK. *BMC Psychol*. 2021;9(1):60. doi:10.1186/s40359-021-00565-y

3. Tagami U, Imaizumi S. No Correlation Between Perception of Meaning and Positive Schizotypy in a Female College Sample. *Front Psychol*. 2020;11. doi:10.3389/fpsyg.2020.01323
